# Splenic Capsule Injury: A Rare Complication of Sigmoid Volvulus

**DOI:** 10.7759/cureus.41118

**Published:** 2023-06-28

**Authors:** Rrezane Miftari, Joshua Gazzetta

**Affiliations:** 1 General Surgery, Saba University School of Medicine, The Bottom, BES; 2 General Surgery, Saint Luke’s Hospital, Kansas City, USA

**Keywords:** case report, hemoperitoneum, sigmoid volvulus, spleen capsule, splenic laceration, splenic injury

## Abstract

Sigmoid volvulus can lead to life-threatening complications. We report a splenic capsule avulsion injury requiring laparotomy as a complication of sigmoid volvulus. A 73-year-old woman was admitted with abdominal distension, rigidity, and tenderness. CT abdomen revealed a splenic injury and hemoperitoneum along with possible sigmoid volvulus. The patient required an emergent exploratory laparotomy due to an acute abdomen and hemodynamic instability. A left colectomy, on-table sigmoidoscopy, hemostasis of the spleen, and temporary abdominal closure were performed. She required subsequent operations for end colostomy and abdominal closure. We establish that splenic lacerations are rare but life-threatening complications of sigmoid volvulus. Careful assessment of the spleen on abdominal imaging and clear visualization of the spleen during sigmoid volvulus surgery is recommended for early recognition and prompt management of splenic injury.

## Introduction

Splenic injuries are common abdominal injuries typically provoked by blunt or penetrating abdominal trauma. Splenic capsule injuries from indirect trauma to the spleen are now well-recognized complications of colonoscopies [1], colorectal surgeries [2,3], and gynecologic oncology surgeries [4]. The spleen is a highly vascularized organ and, when injured, can quickly lead to hemodynamic instability. Unrecognized splenic hemorrhage is life-threatening and makes early intervention crucial.

To our knowledge, a splenic capsule injury and subsequent hemorrhage as a complication of sigmoid volvulus have not yet been reported. Sigmoid volvulus refers to the twisting of the sigmoid colon around its mesentery. This twisting of the colon can potentially cause avulsion on the splenic capsule from increased traction on the splenocolic ligament or pre-existing intrabdominal adhesions. Here we report a rare case of splenic lacerations and hemoperitoneum as an unusual but life-threatening complication of sigmoid volvulus.

## Case presentation

**Figure 1:**
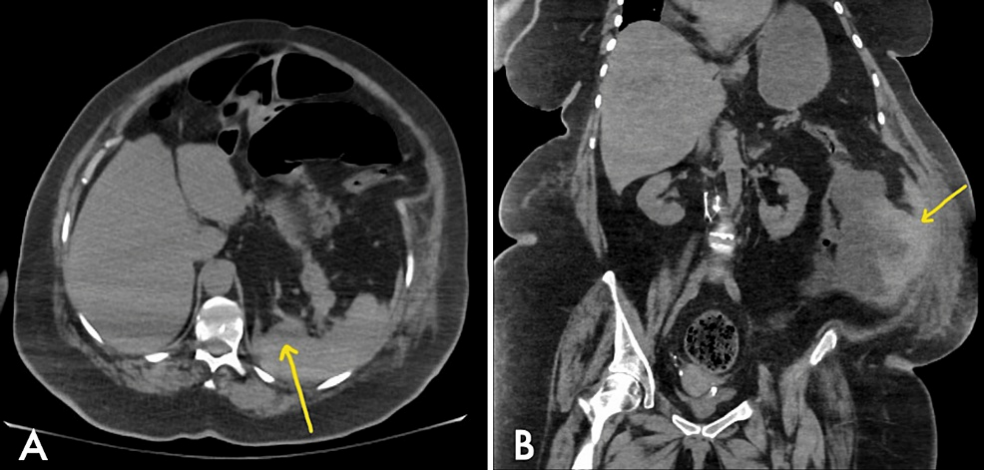
Abdomen/pelvis computed tomography scan: axial view with the yellow arrow showing splenic injury (A), and coronal view with the yellow arrow showing hemoperitoneum (B)

**Figure 2:**
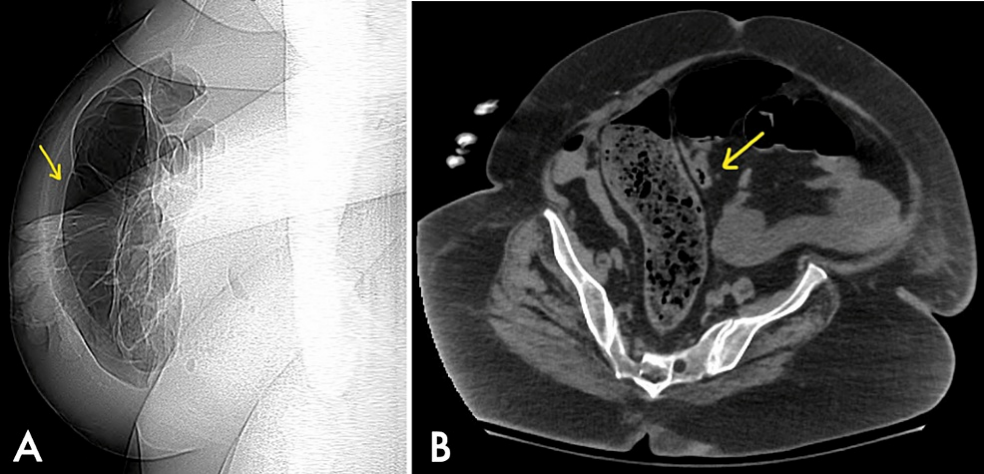
Abdomen/pelvis computed tomography scan: scout film with the yellow arrow showing marked bowel dilation (A), and axial view with the yellow arrow pointing at the transition area of sigmoid volvulus (B)

The patient is a 73-year-old female who was transferred to our hospital for surgical evaluation after a CT abdomen/pelvis revealed hemoperitoneum and possible splenic injury. Upon arrival, the patient was in respiratory distress with labored breathing requiring intubation and mechanical ventilation. Shortly after intubation, the patient became hypotensive, with a blood pressure of 81/61 mmHg and a heart rate of 97 beats per minute, requiring fluid resuscitation and vasopressor support. The patient remained afebrile with a temperature of 37°C. The physical exam demonstrated a severely distended, rigid, tympanitic abdomen with peritoneal signs. The patient had a white blood cell count of 12.28 TH/uL (N: 4.00 - 11.00 TH/uL), hemoglobin of 9.7 g/dL (N: 12.0 - 15.0 g/dL), and platelet count of 257 Th/uL (N: 140 - 400 Th/uL). The chemistry panel was unremarkable. In light of the physical exam, hemodynamical instability, CT abdomen/pelvis imaging, splenic hemorrhage (Figure 1), and possible sigmoid volvulus (Figure 2) were suspected. The patient was taken to the operating room for emergent exploratory laparotomy.

**Figure 3:**
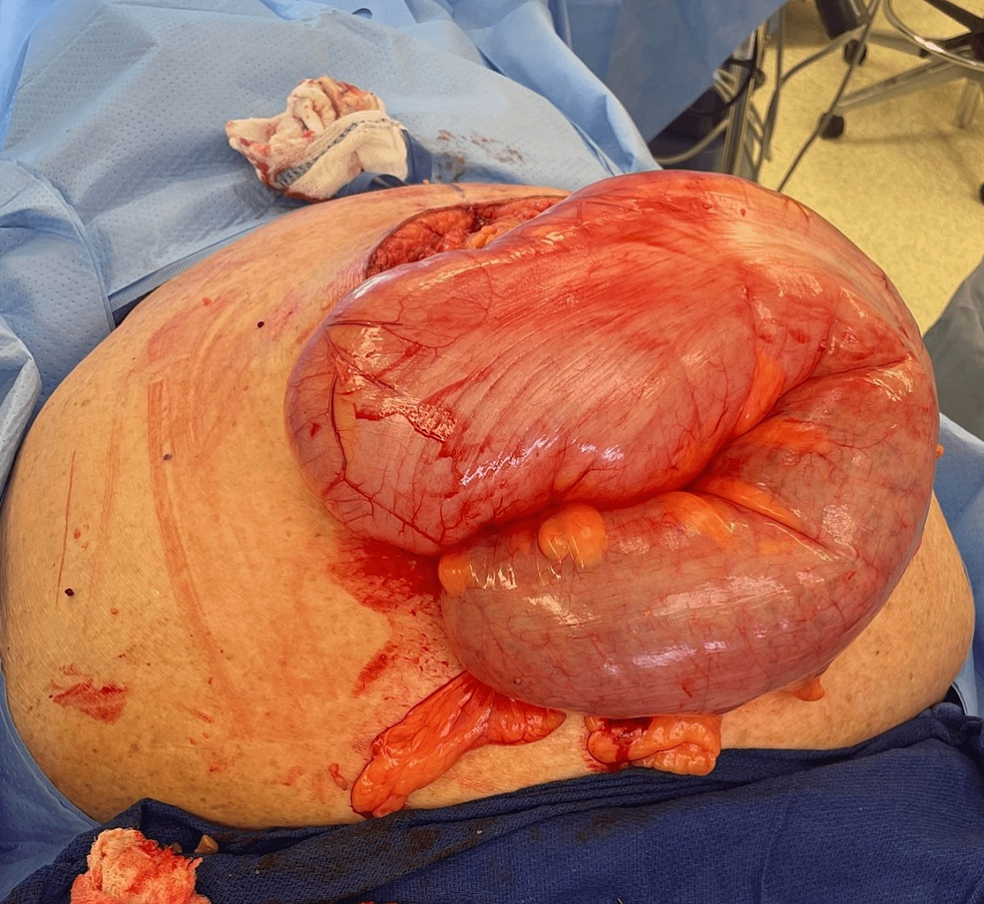
Intraoperative finding of the eviscerated sigmoid colon.

The sigmoid colon was readily eviscerated after a midline celiotomy entry to the abdomen (Figure 3). One liter of blood was immediately evacuated. Extensive adhesions and a desmoplastic reaction with scarring were found throughout the abdomen, which prompted lysis of adhesions for over two hours. The proximal descending colon to the rectum was significantly attenuated and dilated to 15 cm. The left colon was twisted in adhesions which were lysed. A left colectomy and takedown of splenic flexure were performed. The spleen was visualized and found to have two lacerations on the inferior pole secondary to traction from the omental adhesions and splenocolic ligament due to the twisted colon. One of the capsular tears was 1 cm and actively bleeding; it was cauterized, and a hemostatic agent was placed. An on-table sigmoidoscopy was performed since the rectum was also dilated throughout. A mass or stricture was not appreciated, and the scope could traverse the entire sigmoid colon. The patient was left in discontinuity, and a temporary closure device was left in place, given the patient's hemodynamic instability. The patient returned to the operating room after resuscitation. Her abdomen was re-explored, and the fascia could not be reapproximated secondary to tension; thus, a Wittman patch was placed. On the third re-exploration, a drain and gastrojejunostomy tube was placed, a colostomy was created, and the fascia was successfully closed. An ileus and a superficial surgical site infection complicated the remainder of her hospital stay. The patient was discharged in stable condition to a long-term care facility after 20 days of hospitalization. Pathology revealed acute serositis, serosal adhesions, and hemorrhage within the peri colonic adipose tissue; no dysplasia or malignancy was identified.

## Discussion

Splenic injuries are common abdominal injuries with a high risk of mortality due to the rich vascular supply and the anatomic location of the spleen. The consistency of the splenic capsule could also contribute to the increased susceptibility to injury [5]. To our knowledge, splenic capsule injury and hemorrhage associated with sigmoid volvulus have not been previously reported. The clinical presentation of a splenic capsule injury is similar regardless of etiology. In the case of undetected bleeding, hypovolemic shock will quickly develop, which was seen in our patient. We recommend a high level of suspicion of spleen involvement upon diagnosis of sigmoid volvulus when patients present with hemoperitoneum. CT scan of the abdomen is the diagnostic imaging of choice to detect splenic injury and aid in management. In addition, frequent physical exams and haematologic indices should be obtained. Managing splenic injury highly depends on the patient's hemodynamic status. Operative management is indicated in the presence of hemodynamic instability or peritoneal signs [5,6].

The mechanism of splenic capsule injury from sigmoid volvulus has not been well elucidated. We suspect that excessive traction in the splenocolic ligament led to splenic capsule avulsion. The extensive intrabdominal adhesions in our patient may likely have contributed to the injury. Similar mechanisms of injury have been proposed in several iatrogenic causes of splenic injuries. It is well established that there is a small risk of splenic injury post-colonoscopy. The tension applied in the splenocolic ligament during the procedure, and the adhesions between the spleen and the colon remain the leading explanations for injuries to the spleen during colonoscopy [7-9].

Interestingly, a histological report of splenic rupture that occurred after a laparoscopic cholecystectomy supported the idea that stretching the splenic capsule led to a subcapsular hematoma. The authors stated that the adhesions between the splenic capsule and parietal peritoneum may also contribute to these changes [10]. The mobilization of the splenic flexure is one of the main risk factors for injury to the spleen during colon surgeries [3,11,12]. Intraabdominal surgeries can cause injuries to the spleen in the form of a splenic tear, laceration, and, less commonly, splenic rupture [12]. In addition to manipulation of the colon and intrabdominal adhesions, other contributing factors for splenic injury include inflammation from abdominal infections, increased therapeutic colonoscopies, and redundant colon [13].

On the other hand, congenital anomalies of the colon resulting from malrotation or defective fixation of the bowel during development could increase the risk of developing a volvulus [14]. How congenital anomalies of the colon affect the spleen is yet to be discerned.

## Conclusions

To our knowledge, this is the first reported case of splenic capsule injury as a complication of sigmoid volvulus. The spleen should be assessed while establishing a diagnosis of sigmoid volvulus on a CT scan of the abdomen and carefully examined during exploratory laparotomy for sigmoid volvulus if hemoperitoneum is present. Early recognition of such injury and prompt management are critical for positive outcomes.
